# Summary of taxonomy changes ratified by the International Committee on Taxonomy of Viruses (ICTV) – General taxonomy proposals, 2025

**DOI:** 10.1099/jgv.0.002116

**Published:** 2025-07-25

**Authors:** F. Murilo Zerbini, Anya Crane, Jens H. Kuhn, Peter Simmonds, Elliot J. Lefkowitz, ICTV Taxonomy Summary Consortium, E.M. Adriaenssens

**Affiliations:** 1 Dep. de Fitopatologia/BIOAGRO, Universidade Federal de Viçosa, Viçosa, Brazil; 2 Integrated Research Facility at Fort Detrick (IRF–Frederick), Division of Clinical Research, National Institute of Allergy and Infectious Diseases, National Institutes of Health, Fort Detrick, Frederick, Maryland, USA; 3 Nuffield Department of Medicine, University of Oxford, Oxford, UK; 4 Department of Microbiology, University of Alabama at Birmingham (UAB), Birmingham, Alabama, USA

## Abstract

During the 56th annual meeting of the International Committee on Taxonomy of Viruses (ICTV), held in Bari, Italy, in August 2024, two technical proposals were presented. The first called for amended versions of accepted taxonomic proposals to be named in such a way to ensure that they are readily accessible on the ICTV website (2024.001G). The second proposed a substantial reformatting of the ICTV statutes and codes to produce a more unified text after the numerous changes made to both documents in previous years (2024.002G). Finally, the ICTV Executive Committee (EC) nominated Professor Stuart Siddell as a Life Member of the ICTV for his work over four decades on virus taxonomy, including 16 years as a member of the EC (2024.003G).

## Introduction

Three taxonomy proposals of a general nature were approved by the International Committee on Taxonomy of Viruses (ICTV) in 2024. Two technical proposals were presented: the first proposed an updated and uniform system for naming proposals for error correction of accepted taxonomic proposals to ensure that they are readily accessible on the ICTV website (2024.001G). The second proposed to reformat the ICTV statutes and codes to produce a more unified text after numerous piecemeal changes to both documents made in previous years (2024.002G). The revised code and statutes are available at https://ictv.global/about/code and https://ictv.global/about/statutes.

The ICTV Executive Committee (EC) proposed that Professor Stuart Siddell be made a Life Member of the ICTV (2024.003G). Stuart received a B.Sc. degree in 1972, a B.Sc. degree (Hons, Class I) in Botany from the University of Liverpool, UK, in 1973, a Ph.D. in Biological Sciences from the University of Warwick, UK, in 1976, and a Dr. rer. nat. habil. degree (Biochemie) from the University of Würzburg, Germany. After positions at the Imperial Cancer Research Fund, UK, and the University of Würzburg, Germany, Stuart continued his career at the University of Bristol as a Professor of Virology from 1992 until his retirement in 2015, when he became an Emeritus Professor of Virology. His research focused on coronaviruses and played a role in the discovery of coronavirus discontinuous transcription; the development of reverse genetic systems for human, avian and murine coronaviruses; the elucidation of coronavirus protein structures; and constructing a genetic map of coronavirus replicase protein function [1,2].

Stuart began his work with the ICTV in 1982 on the *Coronaviridae* Study Group, serving as chair and subsequently as a member until 2000. He also served as an ICTV National Member for the United Kingdom from 1996 to 2011. Stuart served on the EC as an elected member from 2008 to 2014, Chair of the Animal dsRNA and ssRNA(−) Viruses Subcommittee from 2014 to 2017, and then as Vice-President until 2023. Stuart was also Editor-in-Chief of the ICTV 10th Report, helping to guide the transition of this key ICTV product from a hard-copy book to an online web-based resource [3]. Overall, Stuart provided over 40 years of service to the ICTV, including 16 years on the EC.

During his tenure on the EC, Stuart was a key participant in several significant changes to the processes, methods and intellectual underpinning of the approaches used by the ICTV to guide the classification of viruses and name the resulting taxa. These changes included the creation of higher rank taxonomic categories that support the deeper evolutionary classification of viruses [4], the establishment of evolutionary history as a key guiding principle underlying the ICTV taxonomy [5] and consultation on the use of binomial names for virus species [6] that paved the way for their recent adoption by the ICTV.

He was also responsible for creating and maintaining the Virus Metadata Resource (VMR), a spreadsheet that, for each species, lists an exemplar virus, along with a suggested abbreviation, a GenBank accession for the genome sequence, and general details about genome composition and type of host (https://ictv.global/vmr). The VMR is now a key resource in virology. More recently, Stuart has been responsible for collating information on the etymology of virus taxa for ranks at the family level and above (https://ictv.global/taxonomy/etymology); curating a collection of virion diagrams organized by family (https://ictv.global/virion-diagrams); and assembling a table of virus genome, virion properties and type of host for all virus families (https://ictv.global/virus-properties).

## Main Text

## Contents

2024.001G.Name_format_of_Taxonomy_Proposal_Corrections
2024.002G.ICVCN_and_Statutes_harmonization
2024.003G.Nomination_Stuart_Siddell_as_Life_Member

## 2024.001g.Name_format_of_Taxonomy_Proposal_Corrections

**Title:** Name format for expedited corrections of taxonomy proposals

**Authors:** Simmonds P (Peter.Simmonds@ndm.ox.ac.uk), Zerbini M, Lefkowitz EJ

**Summary**

**Brief description of current situation**
The ratified taxonomy proposal (TP) 2020.002G.Expedited_error_correction describes how errors in the proposal spreadsheet may be corrected with approval from the ICTV President, Data Secretary and Proposals Secretary without the need to re-submit a corrected TP in the next ICTV cycle. However, it was not specified how the name used for the correction TP should be formatted.
**Proposed changes**
We propose that the TP code will be suffixed with an X, and the name suffixed with the term ‘_Error_Correction’.
**Justification**
It is useful to use a standard format that unambiguously links the original and the
correction TPs together.

*Source/full text: https://ictv.global/ictv/proposals/2024.001G.Name_format_of_Taxonomy_Proposal_Corrections.docx

## 2024.002G.ICVCN_and_Statutes_harmonization

**Title:** Revise the ICTV Statutes and the ICVCN

**Authors:** Zerbini M (zerbini@ufv.br), Lefkowitz EJ (elliotl@uab.edu), Crane A, Kuhn J (jenshkuhn@comcast.net)

**Summary**

**Brief description of current situation**
In recent years, numerous individual and several bulk changes were made to the ICTV International Code of Virus Classification and Nomenclature (ICVCN) and the ICTV Statutes. These changes left both documents somewhat in disarray, including problems associated with terminology consistency, orthography and grammar, clarity and application examples.
**Proposed changes**
We propose several changes to both documents to improve their qualities.

*Source/full text: https://ictv.global/ictv/proposals/2024.002G.ICVCN_and_Statutes_harmonization.docx

## 2024.003g.Nomination_Stuart_Siddell_as_Life_Member

**Title:** Nomination of Stuart Siddell as a Life Member of the ICTV

**Authors**: Lefkowitz EJ (elliotl@uab.edu), Zerbini M

**Summary**

Given Professor Stuart Siddell’s long service as a member of ICTV Study Groups, Subcommittees and the EC, given his key role in helping to define and modernize the guiding principles used to create the virus taxonomy, and given his calm demeanour, comforting presence and unwavering friendship to members of the EC over many years, we hereby nominate Stuart Siddell as a Life Member of the ICTV.

*Source/full text: https://ictv.global/ictv/proposals/2024.003G.Nomination_Stuart_Siddell_as_Life_Member.docx

## Abbreviations

EC: Executive Committee
ICTV: International Committee on Taxonomy of Viruses
ICVCN: International Code of Virus Classification and Nomenclature
TP: Taxonomy proposal
VMR: Virus Metadata Resource
